# Effect of Sardine and Sprat Thermal Processing on Intestinal Integrity and Macrophage Activation In Vitro

**DOI:** 10.3390/foods14213754

**Published:** 2025-10-31

**Authors:** Ivo Doskočil, Barbora Lampová, Petr Šmíd, Mariola Drozdowska, Aneta Kopeć

**Affiliations:** 1 Department of Microbiology, Nutrition and Dietetics, Faculty of Agrobiology, Food and Natural Resources, Czech University of Life Sciences Prague, Kamycka 129, 165 00 Prague-Suchdol, Czech Republic; lampova@af.czu.cz; 2 Department of Chemistry, Faculty of Agrobiology, Food and Natural Resources, Czech University of Life Sciences Prague, Kamycka 129, 165 00 Prague-Suchdol, Czech Republic; smidp@af.czu.cz; 3 Department of Human Nutrition and Dietetics, Faculty of Food Technology, University of Agriculture in Kraków, 31-425 Kraków, Poland; mariola.drozdowska@urk.edu.pl (M.D.); aneta.kopec@urk.edu.pl (A.K.)

**Keywords:** omega-3 fatty acid, intestinal barrier, nitric oxide, tumor necrosis factor, INFOGEST, RAW264.7, Caco-2

## Abstract

Small pelagic fish, such as sardines and sprats, are an affordable and nutritionally rich source of omega-3 fatty acids and bioactive peptides. While their nutritional value is well established, the impact of standard household cooking methods on their immunomodulatory potential and effects on intestinal integrity remains poorly understood. Fish were prepared using five culinary techniques (raw, boiled, steamed, baked, and fried), digested via the INFOGEST protocol, and applied at 1% concentration in a Caco-2 co-culture model combined with lipopolysaccharide-stimulated RAW264.7 macrophages. NO and TNF-α production, and epithelial permeability were assessed. Steamed sardines induced the highest NO levels (122%) in activated macrophages, while raw sardines inhibited NO production (73%). Baked sardines and raw sprats triggered higher TNF-α production (>400 pg/mL). Boiled sardines and baked sprats caused the strongest disruption of epithelial permeability (>13%), whereas steamed sardines and raw sprats preserved barrier integrity (<11%). Notably, digested baked and fried fish preserved suppressive effects on NO and TNF-α even after translocation across the epithelial layer. Culinary processing significantly modulates the bioactivity of fish. In general comparison, steaming is gentler than dry heat cooking methods, as it better preserves anti-inflammatory effects and barrier-promoting properties. These findings highlight the relevance of cooking practices in modulating the functional benefits of fish consumption.

## 1. Introduction

Small pelagic fish, such as sardines (*Sardina pilchardus*) and sprats (*Sprattus sprattus*)**,** are widely consumed due to their availability and high nutritional value. They provide high-quality proteins, bioavailable micronutrients and long-chain omega-3 polyunsaturated fatty acids (PUFAs). These compounds have been consistently linked with beneficial effects on cardiovascular and metabolic health, which underpins dietary recommendations to increase oily fish intake [1].

Oily fish represent one of the richest natural sources of immunomodulatory compounds. Omega-3 fatty acids exert effects across both innate and adaptive immunity: they remodel cell membranes, attenuate T-cell activation, and support the development of regulatory T-cells. Additionally, they reduce secretion of pro-inflammatory cytokines, such as tumor necrosis factor α (TNF-α) and interleukin 1β (IL-1β), while promoting anti-inflammatory mediators, such as interleukin 10 (IL-10), through mechanisms involving nuclear factor κB (NF-κB) suppression and peroxisome proliferator-activated receptor γ (PPAR-γ) activation [2,3,4,5,6]. They also serve as precursors for specialized lipid mediators, including resolvins and protectins, which actively promote the resolution of inflammation [2,6,7].

In the gastrointestinal tract, omega-3 fatty acids contribute to maintaining homeostasis by strengthening intestinal barrier integrity, reducing circulating lipopolysaccharide (LPS) levels. Clinical and experimental studies consistently link higher omega-3 intake with improved barrier function and attenuated permeability during inflammatory stress [6,8,9]. Meta-analyses confirm that supplementation with omega-3 fatty acids lowers circulating TNF-α levels in both human and animal studies [2,4,8,10]. They also reduce inducible nitric oxide (NO) synthase (iNOS) expression and NO production in intestinal cells, although the magnitude of these effects depends on the type of fatty acid and experimental conditions [11,12].

Macrophages represent a particularly relevant model for studying these processes. They are key regulators of immune balance and barrier maintenance [13]. However, comparative studies have shown important differences between murine macrophage cell lines, such as RAW264.7 and human-derived macrophages, highlighting challenges in translating mechanistic findings [14,15]. Even so, macrophages provide a useful system to explore omega-3-mediated changes in inflammatory signaling. Eicosapentaenoic and docosahexaenoic acid can exert context-dependent effects; in unstimulated RAW264.7 macrophages, they may enhance NO synthesis, whereas under LPS-induced inflammatory conditions, they suppress NO output. This dual behavior reflects a capacity to either activate or resolve immune responses depending on cellular state, involving pathways, such as mitogen-activated protein kinase (MAPK) and NF-κB [16,17].

In practice, small pelagic fish are consumed primarily after culinary processing. Heat treatments, such as baking, steaming, frying, or roasting, markedly influence their biochemical composition. Gentle moist-heat methods, such as steaming, tend to better preserve functional integrity of sensitive compounds compared to dry-heat techniques, such as roasting [18]. Nevertheless, processed fish proteins can still retain bioactivity. For instance, protein hydrolysates derived from sardines reduced reactive oxygen species, NO production, and pro-inflammatory cytokine secretion in co-culture models, thereby protecting intestinal barrier integrity [19].

Although substantial evidence supports the immunomodulatory and anti-inflammatory roles of omega-3 fatty acids and fish-derived peptides, it remains unclear whether these effects are preserved following typical household cooking. Most available studies focus on the nutritional composition of fish or on purified compounds, but little is known about how real-world culinary practices impact their biological activity, particularly in immune models.

The aim of this study was therefore to determine whether different cooking methods applied to sardines and sprats influence their ability to modulate inflammatory responses and support intestinal barrier function. We focused specifically on NO production and TNF-α expression in an in vitro macrophage model to provide insight into how everyday culinary choices may shape the bioactivity of small pelagic fish.

## 2. Materials and Methods

### 2.1. Sample Preparation

Sardines and sprats were purchased from a commercial food retailer in Poland. Fish samples were thermally processed by boiling, steaming, baking, and frying, while one sample from each species was left in its raw state. The exact specifications of their modification are provided by Skoczylas et al. [1]. All samples were then lyophilized, homogenized, and transported to the Czech University of Life Sciences for further analysis.

### 2.2. Sample Digestion

Ground samples were digested using the static in vitro digestion model INFOGEST 2.0 [20]. Following the protocol described in detail by Doskocil et al. [21]. Briefly, samples underwent oral, gastric, and intestinal phases of digestion, followed by centrifugation and sterile filtration. The resulting supernatants were stored at −80 °C until further use.

### 2.3. Cultivation

The human intestinal Caco-2 cell line (ATCC HTB-37; ATCC, Manassas, VA, United States) was used for the intestinal permeability assay. Cells were cultured in DMEM (Dulbecco’s Modified Eagle Medium) supplemented with 10% fetal bovine serum (FBS), 1% sodium pyruvate, 1% sodium bicarbonate, 1% non-essential amino acids (all from Biowest, Nuaillé, France), and 1% ZellShield (Minerva Biolabs, Berlin, Germany). Cells were maintained at 37 °C in a humidified atmosphere containing 5% CO_2_, with regular medium changes until reaching 80% confluence.

The murine RAW264.7 macrophage cell line (ATCC TIB-71; ATCC) was grown in complete RPMI medium supplemented with 10% FBS, 1% glucose, 1% glutamine, and 1% non-essential amino acids (all Biowest). At 80% confluence, cells were mechanically detached, centrifuged (10 min, 170× *g*), and resuspended in fresh complete medium. A suspension of 1 × 10^6^ cells was seeded into culture flasks containing 10 mL of medium and incubated for 48 h at 37 °C in a humidified CO_2_ atmosphere.

### 2.4. Permeability Assay

A modified version of the method by Hubatsch et al. [22] was used. Caco-2 cells were pre-cultured for 2 weeks in supplemented DMEM, as described above. Cells were seeded into filter inserts (with growth area *A* = 0.33 cm^2^, pore size 0.4 µm; VWR International, Střibrná Skalice, Czech Republic) at a density of 2.6 × 10^5^ cells/cm^2^. The basolateral chamber was filled with 750 µL of DMEM and incubated at 37 °C with 5% CO_2_ for 6 h. Non-adherent cells were removed and replaced with 250 µL of fresh DMEM on the apical side. The medium was replaced every other day during the first week and daily during the second week. The final medium exchange was performed 16 h before the experiment.

After 21 to 25 days of culture, filter inserts were washed three times with pre-warmed Hanks’ Balanced Salt Solution (HBSS) (Sigma-Aldrich Prague, Czech Republic) (37 °C, pH 7.4), and transepithelial electrical resistance (TEER) was measured at 37 °C using an EVOM3 (World Precision Instruments Germany GmbH, Friedberg, Germany). Only inserts with TEER values above 600 Ω⋅cm^2^ were used.

Lucifer Yellow (LY; 25 µM, Sigma-Aldrich) was added to the apical (donor) side (donor volume *V*_d_ = 250 µL); the basolateral (receiver) chamber contained HBSS (receiver volume *V_r_* = 750 µL). Plates were maintained at 37 °C with orbital shaking (250 rpm) throughout the assay. Receiver samples were collected at 0, 30, 60, 120, 180 and 240 min; at each time point, 100 µL was removed from the receiver side and immediately replaced with pre-warmed HBSS to keep *V_r_* constant. Fluorescence was measured at excitation/emission wavelengths of 480/530 nm on a Tecan Spark^®^ multimode reader (Tecan Trading AG, Männedorf, Switzerland). LY concentrations were obtained from a calibration curve prepared in HBSS (blank-subtracted; linear range only).

To correct for serial sampling, the cumulative amount in the receiver was calculated as

$$Q_{n}=V_{r}C_{n}+V_{s}\sum_{i=1}^{n-1}C_{i}$$

(V_s_ = 100 µL; C_i_ are measured receiver concentrations). Apparent permeability was derived from the slope of *Q (t)*: $Papp={\left(AC_{0}\right)}^{-1}\frac{dQ}{dt}$, where $C_{0}\;$ is the initial donor concentration. Data are reported as mean ± SD from inserts per condition.

For the Caco-2/RAW264.7 Transwell co-culture, RAW264.7 macrophages were seeded into 24-well plates at 2 × 10^5^ cells/well in antibiotic-free RPMI + 5% FBS and allowed to adhere overnight (24 h) at 37 °C and 5% CO_2_. Caco-2 cells were cultured as previously described. On the day of co-culture, both apical and basolateral compartments contained antibiotic-free DMEM:RPMI (1:1) + 5% FBS. LPS (1 µg/mL; Sigma-Aldrich) was added to the basolateral compartment to stimulate RAW264.7. The apical compartment received 1% (*v*/*v*) digested sample. Co-culture proceeded for 24 h at 37 °C and 5% CO_2_ without shaking. This was a contact-free co-culture (0.4 µm pores). After incubation, samples were taken from the basolateral compartment for analysis of inflammatory markers (NO and TNF-α). The procedure for determining them is described below.

### 2.5. NO Production

RAW264.7 macrophages were seeded into 96-well plates at a density of 2.5 × 10^5^ cells per 100 µL and incubated for 2 h. After incubation, digested samples (0.009–20%) and a control digestive enzyme mixture were added, followed by 1 µg/mL of LPS. A negative control (without LPS) was also included. The final volume per well was adjusted to 200 µL. Plates were incubated for 24 h at 37 °C in a humidified atmosphere containing 5% CO_2_. After incubation, the plates were centrifuged (5 min, 170 × g), and 50 µL of supernatant was transferred to a new 96-well plate. Subsequently, 50 µL of Griess reagent (Sigma-Aldrich) was added. The mixture was incubated for 10 min at 37 °C, after which, absorbance was measured at 540 nm on Tecan Spark^®^ multimode reader. Values were corrected by the reagent/matrix blank measured on the same plate. Because the objective was to quantify reduction in the LPS-induced signal, results are reported as percentage of the LPS control on each plate:

$$\%\;LPS=100\;\times \;\frac{\max\left(0,\;A-A_{blank}\right)}{A_{LPS}-\;A_{blank}}$$

Negative values after blank correction were set to zero for plotting (left-censored in statistics).

### 2.6. MTT Cytotoxicity

Following NO determination, MTT (Sigma-Aldrich) solution (1 mg/mL in RPMI) was added to each well, and the plates were incubated for 2 h. The medium was then replaced with DMSO (Lach-Ner, Neratovice, Czech Republic), and absorbance was measured at 495 nm on Tecan Spark^®^ multimode reader. The IC_5_ value, representing 95% cell viability, was calculated using Magellan 3.2 software, and the corresponding concentration was used in subsequent NO and TNF-α inhibition assays.

### 2.7. TNF-α Determination

TNF-α concentrations were determined using a commercial mouse TNF-α ELISA kit (ADI-900-047, Enzo Life Sciences, Lausen, Switzerland) according to the manufacturer’s instructions. Samples collected from the NO assay were stored at −80 °C and thawed on the day of analysis. Standards or samples (50 µL) were pipetted into the wells, followed by 50 µL of diluent buffer. The plate was incubated for 2 h at 21 °C room temperature on an orbital shaker set to 250 rpm.

The wells were washed four times with 300 µL of wash buffer. Antibody solution (50 µL) was added, and the plate was incubated for another 2 h. This was followed by additional washing steps, the addition of 50 µL of conjugate, and a 30-min incubation. After a final wash, 50 µL of substrate solution was added and incubated for 30 min, followed by the addition of 50 µL of stop solution. Absorbance was measured at 450 nm with background correction at 570 and 590 nm, using a Tecan Spark^®^ multimode reader. Data were analyzed using Microsoft Excel.

### 2.8. Statistical Analysis

Data are expressed as mean ± SD from three independent experiments. Statistical significance was assessed using two-way ANOVA followed by Tukey’s post hoc test. A *p*-value < 0.05 was considered statistically significant. Principal component analysis (PCA) was performed using GraphPad Prism 10.6. Each contains four quantitative variables: TNF-α and NO levels before and after permeability. The data were not transformed or standardised prior to analysis. PCA was used to reduce dimensionality and visualize patterns of variability. Component selection was based on a cumulative variance threshold of ≥75%. The first two principal components (PC1 and PC2), which together accounted for 98.59% of the variance, were retained for interpretation. PCA is embedded with Supplementary Material.

## 3. Results

### 3.1. NO and TNF-α Production Following LPS Stimulation

**Figure 1:**
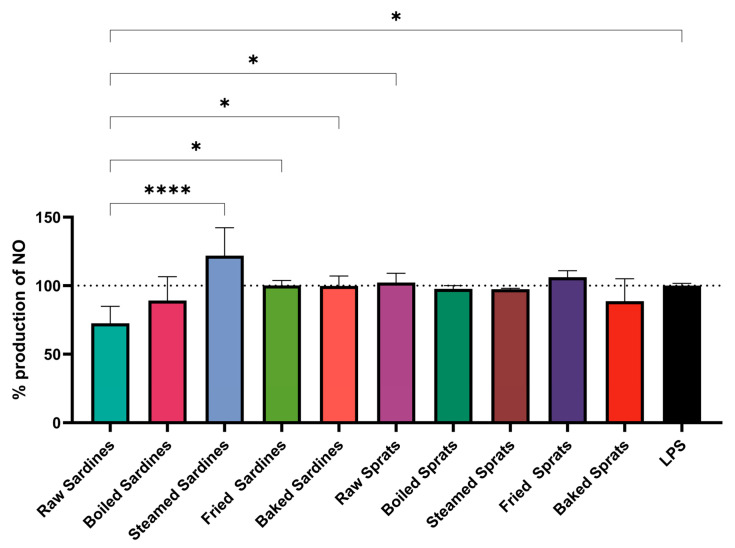
Effect of different culinary treatments of sardines and sprats on nitric oxide (NO) production in LPS-stimulated cells. Data is expressed as percent of the plate-matched LPS control (set to 100%; dotted line) after blank correction. No unstimulated/vehicle (−LPS) control was included on these plates; therefore, the figure reports inhibition relative to LPS rather than absolute values versus basal NO. Values represent mean ± SD. Statistical significance was determined using two-way ANOVA followed by Tukey’s post hoc test; * *p* < 0.05, **** *p* < 0.0001 compared with the LPS control. LPS, lipopolysaccharide.

The method of fish preparation had a clear impact on NO production in LPS-stimulated RAW264.7 macrophages (Figure 1). Steamed sardines induced the highest NO levels (121.96 ± 20.37% relative to the LPS control), with significantly higher values compared with those of raw sardines (*p* < 0.0001), fried sardines (*p* = 0.0327), and most other groups (*p* < 0.05).

Raw sardines had the lowest NO production (72.58 ± 12.29%), which was notably reduced compared with that in the LPS control (*p* = 0.0354). All sprat preparations yielded NO levels comparable to the LPS group, suggesting minimal influence on this parameter.

Further differences were observed between raw and steamed sardines (*p* < 0.0001), baked and steamed sardines (*p* = 0.0374), and between raw sardines and raw sprats (*p* = 0.0163). These results establish both species and preparation methods as relevant factors shaping the NO response profile.

**Figure 2:**
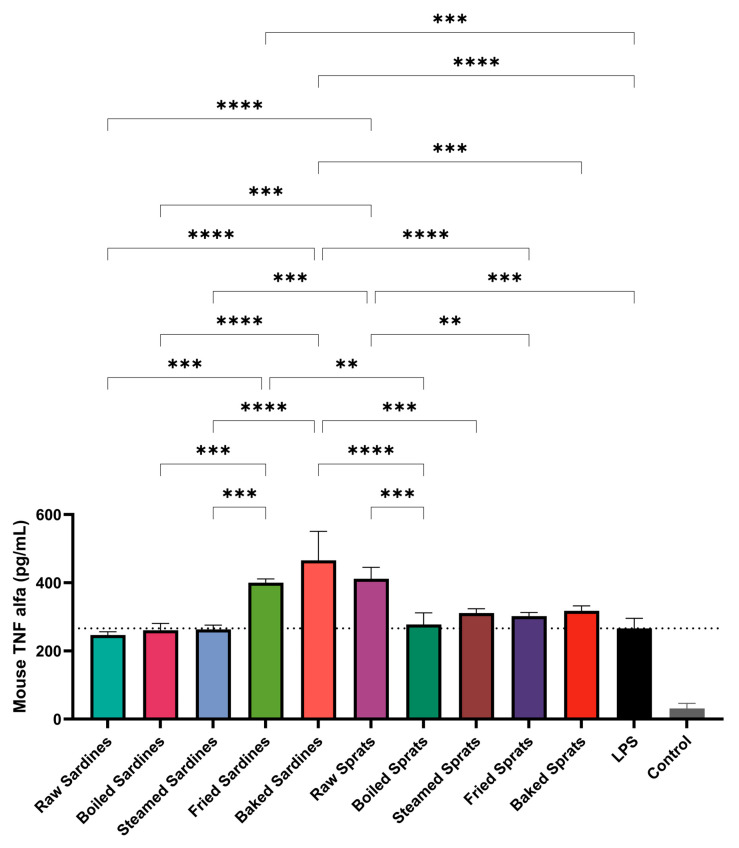
Effect of different culinary treatments of sardines and sprats on TNF-α production in LPS-stimulated cells. Data are expressed as pg/mL and shown relative to the LPS control (100%, dotted line). Values represent mean ± SD . Statistical significance was determined using two-way ANOVA followed by Tukey’s post hoc test; ** *p* < 0.01, *** *p* < 0.001, **** *p* < 0.0001 compared with LPS control. LPS, lipopolysaccharide; NO, nitric oxide; TNF, tumor necrosis factor.

TNF-α levels were also significantly affected by the fish preparation method (Figure 2). Baked sardines induced the highest TNF-α production (498.29 ± 35.34 pg/mL; *p* < 0.0001), followed by raw sprats (409.71 ± 27.58 pg/mL; *p* < 0.0001) and fried sardines (400.79 ± 9.05 ng/mL; *p* < 0.0001). All three significantly exceeded the TNF-α levels observed in the LPS control group (273.54 ± 28.17 pg/mL).

Boiled and steamed sardines triggered TNF-α levels comparable to those of the LPS control, suggesting only a minimal inflammatory effect. A similar pattern was observed for sprats: while frying and baking led to moderate increases in TNF-α production, the steamed and boiled preparations maintained levels close to those of the LPS control.

The control showed minimal TNF-α secretion (31.4 pg/mL), confirming the dependence of the inflammatory response on LPS. In particular, raw sprats induced considerable TNF-α release despite the absence of thermal processing, suggesting that intrinsic properties of the species may contribute to its immunostimulatory potential.

Additional pairwise comparisons revealed further differences (see Figure 2). Among sardines, TNF-α levels were significantly higher in baked vs. steamed (*p* < 0.0001), baked vs. raw (*p* < 0.0001), and fried vs. raw sardines (*p* = 0.0002). Steamed sardines also showed reduced TNF-α compared to fried sardines (*p* = 0.0001).

### 3.2. Intestinal Permeability in Caco-2 Co-Cultures

**Figure 3:**
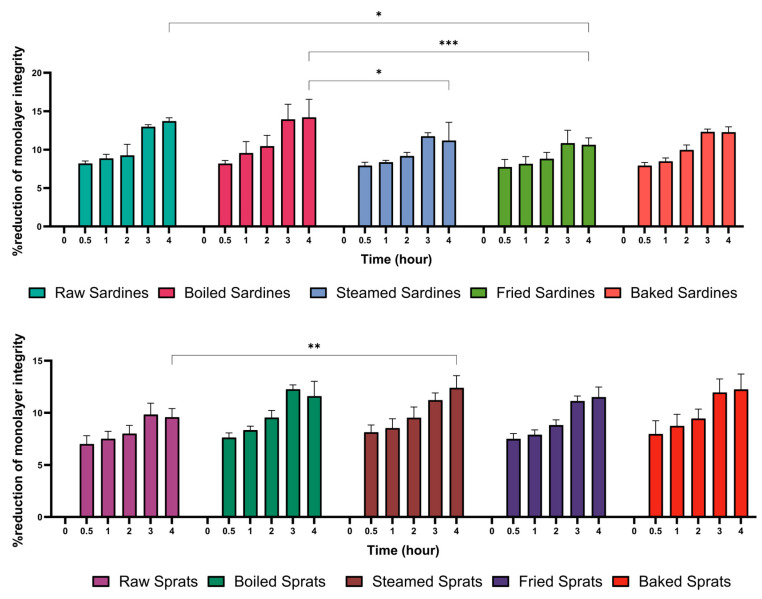
Effect of different culinary treatments of sardines and sprats on epithelial monolayer integrity following. Data are expressed as a percentage of monolayer integrity over time (0–4 h). Values represent mean ± SD. Statistical significance was determined using two-way ANOVA followed by Tukey’s post hoc test; * *p* < 0.05, ** *p* < 0.01, *** *p* < 0.001, compared between groups at each time point.

To assess the effect of fish preparation on intestinal tight junction, paracellular permeability was measured in Caco-2 co-cultures over a 4-h incubation period (Figure 3). All treatments led to time-dependent increases in permeability, with marked differences observed across species and cooking methods.

Among sardines, raw samples induced the strongest disruption (13.72 ± 0.43%), followed by boiled (14.20 ± 2.36%) and baked sardines (12.27 ± 0.69%). Steamed (11.19 ± 2.38%) and fried sardines (10.64 ± 0.89%) caused more moderate permeability increases, typically remaining within the 10–11% range.

Sprats showed a comparable pattern. Baked sprats resulted in the highest increase (12.25 ± 1.47%), while boiled (11.60 ± 1.43%) and raw sprats (9.59 ± 0.82%) showed milder effects, with permeability remaining below 10%.

Statistical comparisons revealed that raw sardines differed significantly from fried sardines (*p* = 0.0057) and raw sprats (*p* < 0.0001). Boiled sardines showed significant differences from steamed (*p* = 0.0088) and fried sardines (*p* = 0.0002), as well as from raw sprats (*p* < 0.0001). Raw sprats also differed from steamed sardines (*p* = 0.0266).

These findings suggest that both fish species and preparation methods influence the degree of intestinal barrier disruption.

### 3.3. Inhibition of NO and TNF-α Production after Permeability (1% Digestate)

**Figure 4:**
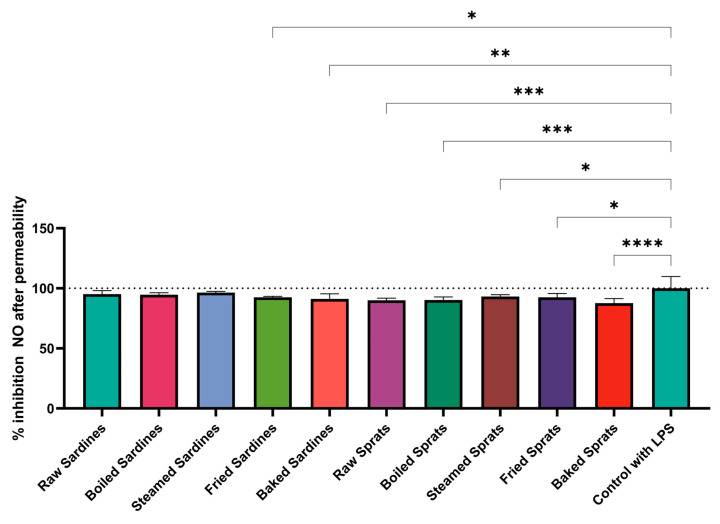
Inhibitory effect of sardines and sprats, prepared using different culinary treatments, on nitric oxide (NO) production following increased epithelial permeability in LPS-stimulated cells. Data are expressed as percentage inhibition relative to the LPS control (100%, dotted line). Values represent mean ± SD. Statistical significance was determined using two-way ANOVA followed by Tukey’s post hoc test; * *p* < 0.05, ** *p* < 0.01, *** *p* < 0.001, **** *p* < 0.0001 compared with the LPS control. LPS, lipopolysaccharide.

To determine whether digested products could still modulate macrophage responses after traversing the intestinal barrier, we re-evaluated NO production in LPS-stimulated macrophages exposed to 1% concentrations of the permeated digests after a 24-h incubation (Figure 4).

Notable reductions in NO levels relative to LPS control were observed for several preparations. Fried sardines reduced NO production to 92.56 ± 0.76% (*p* = 0.0147) and baked sardines to 91.21 ± 4.28% (*p* = 0.0025). Among sprats, raw (90.12 ± 2.52%; *p* = 0.0005), boiled (90.28 ± 2.52%; *p* = 0.0006), steamed (93.15 ± 1.50%; *p* = 0.0301), fried (92.55 ± 3.13%; *p* = 0.0146), and baked preparations (87.73 ± 3.74%; *p* < 0.0001) all led to significant NO inhibition.

These findings indicate that specific cooking methods help retain anti-inflammatory properties in fish-derived digests, even after simulated intestinal absorption.

**Figure 5:**
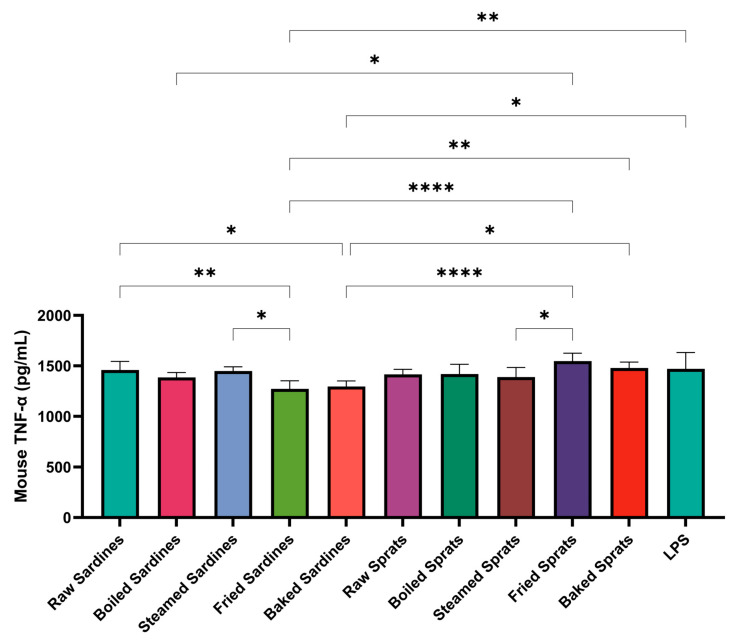
Effect of different culinary treatments of sardines and sprats on TNF-α production in LPS-stimulated cells. Data are expressed as absolute concentrations of TNF-α (pg/mL). Values represent mean ± SD. Statistical significance was determined using two-way ANOVA followed by Tukey’s post hoc test; * *p* < 0.05, ** *p* < 0.01, **** *p* < 0.0001 compared between groups. LPS, lipopolysaccharide; TNF, tumor necrosis factor.

A similar assay was conducted for TNF-α (Figure 5). 

Compared with the LPS-only control (1471.66 ± 160.06 pg/mL), a significant reduction was observed in fried (1272.65 ± 80.28 pg/mL; *p* = 0.0072) and baked sardines (1296.41 ± 54.69 pg/mL; *p* = 0.0295). When compared directly, raw sardines (1458.64 ± 84.69 pg/mL) induced higher TNF-α levels than both fried (*p* = 0.0092) and baked sardines (*p* = 0.0391). Similarly, steamed sardines (1448.44 ± 41.91 pg/mL) showed elevated TNF-α levels compared with those in baked (*p* = 0.0175).

Among sprats, the highest TNF-α levels were recorded in fried samples (1547.97 ± 78.24 pg/mL), whereas steamed sprats produced significantly lower values (1388.99 ± 94.72 pg/mL; *p* = 0.0469). This reduction was also evident when compared with that of both baked (*p* < 0.0001) and fried sardines (*p* < 0.0001).

## 4. Discussion

### 4.1. NO and TNF-α

Based on the data we have collected, the immunomodulatory properties of sardines and sprats are greatly influenced by the heat treatment method. Using a co-culture model of intestinal epithelium and macrophages, we observed that steaming preserved anti-inflammatory activity, while dry-heat methods induced more variable effects. NO and TNF-α levels served as reliable indicators of these immunological shifts, highlighting the sensitivity of fish bioactivity to preparation methods.

Our results show that the method of culinary preparation significantly affects the inflammatory response of LPS-stimulated RAW264.7 macrophages. Steamed sardines had the highest NO production, while raw sardines had the lowest levels. Notably, the higher NO in the steamed group is likely a processing-related effect rather than stronger proinflammatory signalling. Steaming can promote lipid oxidation and aldehyde (e.g., malondialdehyde) protein adduct formation, facilitate post-mortem nitrite to NO conversion under mild heat and low oxygen, and increase protein carbonylation processes that can elevate measurable NO/NOx. Consistent with this interpretation, TNF-α did not increase in the steamed samples, indicating that macrophage activation per se was not augmented [23,24].

This may suggest that moist heat can lead to influencing compounds that stimulate macrophage responses; however, raw samples have limited immunomodulatory effects. Highest TNF-α secretion was in baked sardines, followed by raw sprats and fried sardines, indicating that dry heat processing and fish species can lead to stronger pro-inflammatory response. These differences confirm that both fish species and their method of preparation significantly modulate the immunomodulatory potential of digested samples. A very important finding is that boiled sardines caused the highest NO production among all variants. This effect may be associated with the better preservation of immunologically active components, such as omega-3 fatty acids or bioactive peptides, during moist heat processing [1]. However, it is important to note that increased NO production may not only reflect activation of defense mechanisms but also signal pro-inflammatory macrophage activation. Similarly, the elevated TNF-α secretion observed especially in baked sardines suggests a distinct immunomodulatory profile, potentially resulting from changes in lipid and protein components during dry heat treatment. Our findings are consistent with previous studies featuring the sensitivity of omega-3 fatty acids and peptides to degradation during thermal processing [25]. Moist heat processing, such as steaming, is generally associated with better preservation of structural integrity and bioactivity of these components, while dry heat methods, such as baking or frying, may promote lipid oxidation and protein denaturation [18].

An interesting result is that raw sardines, despite their high content of native omega-3 fatty acids, exhibited low immunomodulatory efficacy. This effect may be related to the presence of intact proteins that are not sufficiently broken down into bioactive fragments capable of interacting with immune cells during digestion. In contrast, thermal treatment may facilitate the release or formation of bioactive peptides, increasing both their bioavailability and immunomodulatory activity.

### 4.2. Permeability

Our results also demonstrated that the differences between preparation methods are significantly reflected in their effects on the intestinal barrier. Data on intestinal permeability underlines the importance of culinary processing. Our findings showed that raw and boiled sardines, as well as baked sprats, significantly disrupted intestinal barrier integrity, whereas steamed and raw sprats held tighter epithelial junctions. This finding is significant given that increased epithelial permeability is connected with the translocation of LPS and subsequent release of pro-inflammatory cytokines, as shown in previous studies [9]. These differences may be explained by the fatty acid composition, which is influenced by thermal processing [1]. Long-chain saturated fatty acids, particularly palmitic (C16:0) and stearic (C18:0) acids, have previously been linked to increased permeability through mitochondrial dysfunction, reduced ATP production, and elevated oxidative stress, leading to weakened tight junctions in intestinal cells [26]. Conversely, certain PUFAs, especially omega-3 and omega-6, support intestinal barrier integrity, reduce permeability, and counteract inflammation- or cytokine-induced barrier disruption [11,27,28].

However, methodological aspects, such as exposure duration in our experiments, should also be considered. For testing intestinal barrier integrity, we used relatively short time intervals, whereas the literature commonly employs longer exposures ranging from 24 to 96 h [29]. Our approach aimed to capture the immediate effect following consumption and thermal processing of the sample, a scenario more reflective of the typical consumer. In contrast, in the co-culture model using Caco-2 intestinal cells and RAW264.7 macrophages, we applied a 24-h exposure, which should be sufficient time for the incorporation of fatty acids into cell membranes and the development of measurable biological effects [30].

Lastly, we examined whether immunomodulatory effects persist after simulated absorption. Importantly, the anti-inflammatory potential of fish digesta persisted even after simulated intestinal absorption. Our results showed that some thermally processed samples, particularly baked and fried sardines and sprats, retained their ability to suppress NO and TNF-α production even after passage through the barrier. This effect may be explained by the presence of heat-generated peptides or lipid mediators that are resistant to digestion and remain biologically active. The literature indeed reports that both synthetic and naturally derived bioactive peptides can effectively suppress TNF-α secretion in immune cells, especially under inflammatory stimuli [31,32,33]. In contrast, there is limited evidence that peptides can influence NO production [34]. Interestingly, raw samples showed only limited immunomodulatory effects after barrier passage, supporting the hypothesis that a certain degree of processing is beneficial for the release of bioactive compounds [35].

### 4.3. Limitations

These findings should also be evaluated in light of our model’s limitations. One of the main limitations of this study is the use of murine macrophages, which do not fully reflect human immune responses, as different receptors are expressed compared to human macrophages, and expression patterns differ between cell lines, which could lead to divergent results [36,37]. Additionally, cytokine levels produced in response to omega-3 fatty acids and inflammatory stimuli (e.g., LPS) may differ between murine and human macrophages, affecting the interpretation of immunomodulatory effects [38,39].

Another limitation is the exclusion of intestinal microbiota, which plays a key role in the metabolism of fish components. Moreover, we did not assess specific peptide sequences or individual lipid mediators, which could provide deeper mechanistic insights into the observed effects.

We choose Caco-2 cells as the epithelial component because they are a widely adopted human intestinal barrier model with robust tight-junction formation, high TEER and well-characterized transporter/enzyme expression, which enables reproducible Transwell co-culture assays and comparisons across studies. At the same time, Caco-2 cells have known limitations: they are colon-derived, require prolonged differentiation, produce little mucus, and their cytokine output and pattern of innate receptors differ from primary intestinal epithelium. These features can lead to tighter barriers and lower basal inflammatory signaling than in vivo. Alternative models each trade off other constraints. HT29-MTX adds a mucus layer but forms a leakier barrier [40]; T84 yields very high TEER but limited differentiation of absorptive functions; primary epithelial monolayers or organoids better recapitulate cell diversity yet bring donor-to-donor variability, cost, and lower throughput [41]. We therefore paired Caco-2 with RAW264.7 macrophages in a contact-free co-culture to capture epithelial–immune crosstalk under LPS challenge but acknowledge that future confirmation in mucus-producing or primary human models would further strengthen generalizability.

Despite these limitations, our results have important implications. They demonstrate that not only the species of fish but also its culinary preparation can significantly influence the resulting immune response. This insight is particularly relevant for the development of dietary recommendations for populations with inflammatory or metabolic disorders. Given the wide availability and popularity of small pelagic fish, there is an opportunity to optimize their preparation techniques to enhance their functional benefits. Future studies should therefore explore the interaction between thermal processing and microbial metabolism, characterize the profiles of bioactive compounds in greater detail, and validate these findings in models relevant to humans.

## 5. Conclusions

This study demonstrated that standard household cooking methods significantly influenced the immunomodulatory potential and effects on the intestinal barrier of small pelagic fish such as sardines and sprats. Among the methods tested, steaming proved most beneficial—reducing nitric oxide and TNF-α production in LPS-stimulated macrophages while preserving epithelial integrity in a co-culture model.

Dry-heat methods, such as baking and frying, preserved some bioactivity. However, they were associated with increased lipid oxidation and a higher pro-inflammatory response. Raw samples showed inconsistent effects, likely due to limited peptide release and the presence of native enzymes that may interfere with cellular signalling.

Importantly, these effects persisted even after the whole simulation of digestion and passage through the intestinal barrier, emphasising the critical role of thermal processing in modulating the biological efficiency of food. Differences observed between sardines and sprats further suggest that fish muscle composition, particularly fatty acid profiles and endogenous antioxidants, plays a key role in determining immunonutritional activity.

In conclusion, these results indicate how culinary processing, mainly wet heat processing, affects the immunonutritional profile of fish. Although these results need to be further confirmed in clinical or in vivo models, they may contribute to developing dietary strategies for populations at risk of inflammatory and metabolic diseases. Future research should focus on identifying the key bioactive compounds, exploring their interactions with the gut microbiota, and validating the observed effects in human-relevant models.

## Data Availability

The original contributions presented in the study are included in the article/Supplementary Materials. Further inquiries can be directed to the corresponding author.

## Supplementary Materials

The following supporting information can be downloaded at: https://www.mdpi.com/article/10.3390/foods14213754/s1, Figure S1: PCA biplot of immune readouts (NO and TNF-α).; Figure S2. Loading plot from PCA of immune markers NO and TNF-α; Figure S3. PCA score plot of samples based on immune markers, NO and TNF-α.

## Abbreviations

The following abbreviations are used in this manuscript:

LPS	Lipopolysaccharide
PCA	Principal component analysis
PUFA	Polyunsaturated fatty acid
TEER	Transepithelial electrical resistance
TNF	Tumor necrosis factor
