# Supplementary material for: Effect of Sardine and Sprat Thermal Processing on Intestinal Integrity and Macrophage Activation In Vitro
Source: Foods. 2025 Oct 31;14(21):3754. doi: 10.3390/foods14213754 (PMC12608999; doi:10.3390/foods14213754)
Supplement: Supplementary file 1 [file foods-14-03754-s001.zip › foods-3896903-supplementary.pdf]

Supplement material PCA

To assess the impact of intestinal barrier passage on the immune response (measured by TNF-α and NO levels), PCA was performed on a dataset of 60 samples, each comprising four variables: concentrations of TNF-α and NO before and after passing through the *in vitro* intestinal barrier.

The first two principal components (PC1 and PC2, see Figure 6) explained 74.81% and 23.78% of the total variance, respectively, accounting for 98.59% overall. Therefore, they were selected for further interpretation.

PCA revealed that the variable TNF-α after contributed strongly and positively to PC1, while TNF-α before showed a negative loading. The variables NO before and NO after were oriented primarily along PC2, suggesting that changes in NO represent a distinct dimension of variability compared to TNF-related changes.

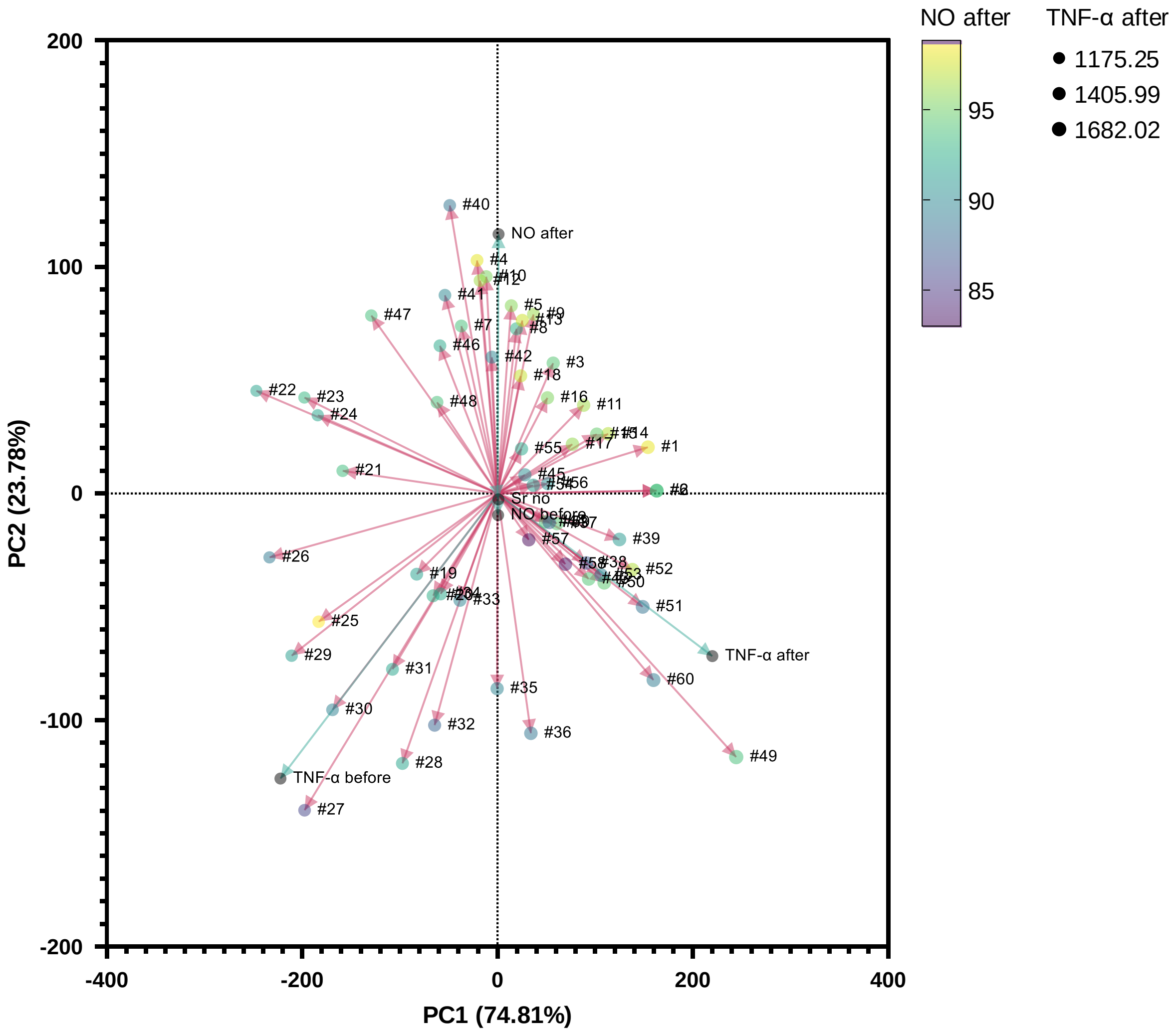

Figure S1. PCA biplot of immune readouts (NO and TNF-α). Points show individual samples; arrows indicate variable loadings (NO before, NO after, TNF-α before, TNF-α after). Axis labels report variance explained (PC1 74.8%, PC2 23.8%). Point colour encodes NO-after; point size encodes TNF-α-after. Samples with higher TNF-α-after cluster to the right along PC1, whereas NO-related variation loads more strongly on PC2. A separation of

samples before vs. after barrier passage is evident primarily along PC1. NO, nitric oxide; TNF, tumor necrosis factor; PCA, principal component analysis.

The loading plot (Figure S2) confirmed that changes in TNF-α had the greatest influence on the PC1 axis, TNF-α after showed a positive loading, while TNF-α before had a negative loading. The variables, NO before and NO after, were located closer to the origin of the PC1 axis and aligned primarily along PC2, indicating that changes in NO levels exhibited a distinct pattern of variability and had a smaller overall contribution to PC1.

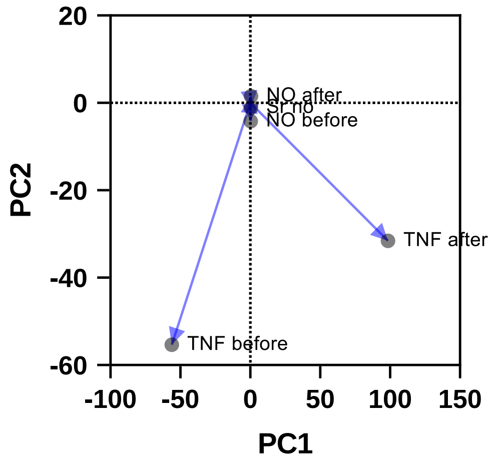

Figure S2. Loading plot from PCA of immune markers NO and TNF-α. The graph displays the contribution of each variable (NO and TNF-α before and after permeability) to the first (PC1) and second (PC2) principal components. TNF after contributes strongly and positively to PC1, while TNF-α before shows a negative loading. The variables NO before and NO after are clustered near the origin along PC1 and oriented more along PC2, indicating lower variance and a different variability pattern compared to TNF-α. NO, nitric oxide; PCA, principal component analysis; TNF, tumor necrosis factor.

The score plot (Figure S3) revealed a clear separation of samples based on their status before and after permeability, particularly along the PC1 axis. Samples with higher TNF-α after levels clustered on the right side and were represented by larger point sizes (reflecting TNF concentration). NO after values were visualized using a color gradient along the PC2 axis.

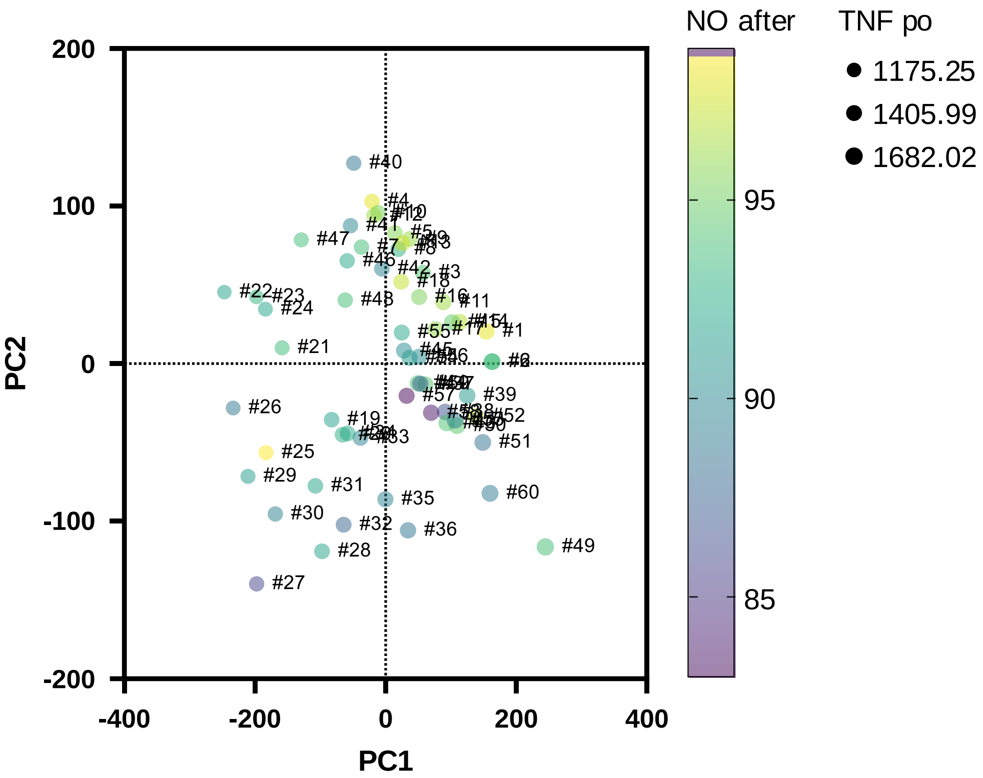

Figure S3. PCA score plot of samples based on immune markers, NO and TNF-α. The plot shows the distribution of individual samples in the space of the first two principal components (PC1 and PC2), which together explain 98.59% of the total variance. Point color represents NO after levels, and point size corresponds to TNF after concentration. Samples with elevated TNF-α after cluster toward the right (higher PC1), while NO after variation is reflected primarily along PC2. NO, nitric oxide; TNF, tumor necrosis factor.

The PCA results indicate that passage through the *in vitro* intestinal barrier has a significant impact on TNF-α levels, whereas changes in NO are less pronounced and orthogonal to TNF-α. PCA enabled effective visualization of immunological response patterns and identification of the key variables contributing to differences between samples.
